# Comparative analyses of the *Hymenoscyphus fraxineus* and *Hymenoscyphus albidus* genomes reveals potentially adaptive differences in secondary metabolite and transposable element repertoires

**DOI:** 10.1186/s12864-021-07837-2

**Published:** 2021-07-04

**Authors:** Malin Elfstrand, Jun Chen, Michelle Cleary, Sandra Halecker, Katarina Ihrmark, Magnus Karlsson, Kateryna Davydenko, Jan Stenlid, Marc Stadler, Mikael Brandström Durling

**Affiliations:** 1 Department of Forest Mycology and Plant Pathology, Swedish University of Agricultural Sciences, Almas Allé 5, Box 7026, SE-750 07 Uppsala, Sweden; 2 Southern Swedish Forest Research Centre, Swedish University of Agricultural Sciences, Sundsvägen 3, Box 49, SE-230 53 Alnarp, Sweden; 3 Dept. Microbial Drugs, Helmholtz Centre for Infection Research, Inhoffenstrasse 7, D-38124 Braunschweig, Germany; 4 Systematic & Evolutionary Botany and Biodiversity group, MOE Laboratory of Biosystem Homeostasis and Protection, College of Life Sciences, Zhejiang University, Hangzhou, Zhejiang China; 5 Ukrainian research Institute of Forestry and Forest Melioration, 62458 Kharkov, Ukraine

**Keywords:** Ash dieback, Viridiol, *Fraxinus excelsior*, Transposable elements, Secondary metabolites, invasive species

## Abstract

**Background:**

The dieback epidemic decimating common ash (*Fraxinus excelsior*) in Europe is caused by the invasive fungus *Hymenoscyphus fraxineus*. In this study we analyzed the genomes of *H. fraxineus* and *H. albidus*, its native but, now essentially displaced, non-pathogenic sister species, and compared them with several other members of Helotiales. The focus of the analyses was to identify signals in the genome that may explain the rapid establishment of *H. fraxineus* and displacement of *H. albidus*.

**Results:**

The genomes of *H. fraxineus* and *H. albidus* showed a high level of synteny and identity. The assembly of *H. fraxineus* is 13 Mb longer than that of *H. albidus’*, most of this difference can be attributed to higher dispersed repeat content (i.e. transposable elements [TEs]) in *H. fraxineus*. In general, TE families in *H. fraxineus* showed more signals of repeat-induced point mutations (RIP) than in *H. albidus*, especially in Long-terminal repeat (LTR)/Copia and LTR/Gypsy elements.

Comparing gene family expansions and 1:1 orthologs, relatively few genes show signs of positive selection between species. However, several of those did appeared to be associated with secondary metabolite genes families, including gene families containing two of the genes in the *H. fraxineus-*specific, hymenosetin biosynthetic gene cluster (BGC).

**Conclusion:**

The genomes of *H. fraxineus* and *H. albidus* show a high degree of synteny, and are rich in both TEs and BGCs, but the genomic signatures also indicated that *H. albidus* may be less well equipped to adapt and maintain its ecological niche in a rapidly changing environment.

**Supplementary Information:**

The online version contains supplementary material available at 10.1186/s12864-021-07837-2.

## Background

Historically, plant disease epidemics have had unprecedented consequences to the global society. Many of the global pandemics and species declines associated with emerging infectious diseases (EIDs) have been a result of human-mediated intercontinental trade and transportation of plant material [1]. In Europe, an epidemic is currently occurring on common ash (*Fraxinus excelsio*r) – a keystone tree species in European temperate forests. The disease, commonly known as ash dieback, was first observed in the early 1990s [2, 3] and has since spread to most European countries dramatically reducing the size of the common ash population and threatening the organisms depending on the tree species for their survival [4]. The causal agent of the disease is the ascomycete fungus *Hymenoscyphus fraxineus* Baral, Queloz, Hosoya [5] that originates in East Asia [6–10] and is considered to be an EID on the European continent. The low genetic variation among European *H. fraxineus* populations indicates a severe genetic bottleneck that is in line with the EID hypothesis [10–12]. The European *H. fraxineus* population was likely founded from the Asian ancestral population by only two different haplotypes [12].

In its native range in east Asia, *H. fraxineus* behaves as an endophyte on its native host *Fraxinus mandshurica*, where it inhabits the living leaves asymptomatically during the growing season. Once the leaves have fallen, it behaves saprotrophically [10, 13]. However, in artificial inoculations of stems of *F. excelsior* and *F. mandshurica*, necrotic lesions occur in both species [14]. Its sister species *Hymenoscyphus albidus*, native to Europe, is non-pathogenic on *F. excelsior* and *F. mandshurica* [15, 16]. *Hymenoscyphus albidus* predominantly acts as a saprotroph on fallen ash leaves and rachises in the leaf litter, possibly preceded by asymptomatic colonization of leaves prior to leaf shed [17]. Both fungal species have a prolonged saprotrophic growth phase after the leaves have fallen, and form pseudosclerotia on the remaining rachises and veins after the leaf blades have disappeared [17]. The pseudosclerotia of both *H. fraxineus* and *H. albidus* act as survival structures during winter, followed by the development of apothecia and spores in the spring [17]. Apart from the differences in the lifestyle of *Hymenoscyphus* species on European ash (*H. fraxineus* appearing pathogenic and *H. albidus* being mainly saprotrophic), the breeding systems of the fungi differ; *H. fraxineus* is outcrossing while *H. albidus* is reported to be self-fertile [18]. The genomes of *H. fraxineus* and *H. albidus* were first published by Stenlid et al. [19]. The study reports broad similarities in the gene content of the two *Hymenoscyphus* species including an extensive repertoire of cell wall-degrading enzymes compared to other Helotialean fungi. This expansion is consistent with their long saprotrophic growth phase degrading leaf rachises [17], or possibly with a necrotrophic lifestyle.

Sequencing of fungal and oomycete genomes has led to an improved understanding of genomic signatures of adaptation in fungi, highlighting the roles of effector proteins, specialized metabolites and repeat/transposable element-rich regions in the genomes in ecological niche colonization [20–22]. Transposable elements (TEs) are impactful drivers of genome evolution and adaption [23, 24]. For instance, comparative genomic studies have revealed that pathogenic fungi tend to have higher TE accumulation compared to non-pathogenic taxa [24]. Transposable element insertions can also cause mating system transition by rearranging the fungal mating-type locus [25–27]. The observation from some filamentous pathogen genomes that genes located in repeat-rich regions tend to evolve faster than those in the rest of the genome, have led to the formulation of the so called two-speed genome hypothesis which proposes that the gene sparse, TE-rich region drives adaptive evolution in fungi [20]. Obviously, the proliferation of TEs can have powerful, even deleterious, effects in their hosts [28, 29]. Although purifying selection or genetic drift may be important determinants to restrict the spread of TEs across the genome [30–32], the harmful potential of TEs has led to the evolution of genome control mechanisms of TE proliferation. Recognition of multicopy DNA sequences, e.g. TEs, can trigger their modification in fungi. Multicopy DNA sequences can undergo cytosine methylation (Methylation Induced Premeiotically, MIP), or Repeat-Induced Point mutations (RIP). RIP introduces point mutations in and adjacent to repetitive sequences that may introduce stop codons or shift DNA methylation patterns [33–35], inhibiting the further expansion of the multicopy elements by preventing the expression of any remnant coding regions [34].

Specialized or secondary metabolism defines metabolic pathways involved in the biosynthesis of a wide variety of small molecules that are characterized by being non-essential for the survival of the organism. Fungal specialized metabolites are associated with e.g. antibiosis, virulence, and/or host-specificity [36] and necrotrophic pathogens are thought to use effector proteins, proteases and carbohydrate-active enzymes, as well as specialized metabolites to colonize their hosts [37]. Consequently, necrotrophic pathogens can harbour an expanded repertoire of specialized metabolite enzymes [36]. Fungal primary and specialized metabolic pathways are often clustered into biosynthetic gene clusters (BGCs). Generally speaking, BGCs may include genes coding for enzymes that catalyse specialized metabolite biosynthesis, transport proteins that export the metabolite out of the cells, and transcription factors that activate expression of the genes in the BGC. BGC repertoires in fungal genomes contribute significantly to ecological niche adaptation of fungi [22, 38]. Consequently, in fungi, genes associated with biosynthesis of secondary metabolites, both those that are found in BGCs and those outside, often show signs of diversification, i.e. duplication and horizontal gene transfer, in and between species [38]. *Hymenoschypus fraxineus* produces a broad spectrum of different specialized metabolites under laboratory conditions [39–46] but their biosynthetic pathways and their role in the interaction with European and Asian ash species is often unclear. For instance, viridiol, which was one of the first compounds to be isolated from cultures of *H. fraxineus*, has phytotoxic activity inducing necrosis on ash tissues in bioassays [39, 47], however viridiol is also produced by *H. albidus* [48], which does not induce dieback on its host. Hymenosetin is another secondary metabolite produced by *H. fraxineus* with wide activity spectrum and could therefore constitute a defence agent that the pathogen secretes to combat competing fungi and bacteria in its natural environment [43].

The overall aim of this study was to use the genome sequences of *H. fraxineus* and *H. albidus* [19] to improve the understanding of the genetic mechanisms that allowed *H. fraxineus* to become an EID on the European continent. We compared the genomic architecture and rates of evolution of *H. fraxineus* and *H. albidus* to those of six other members of Helotiales. Our aim was to test for differences in TE composition, specialized metabolite biosynthesis gene content and selection patterns between the *H. fraxineus* and *H. albidus* genomes, in the context of lifestyle and mating system. We additionally focused on the identification of BGCs of above-mentioned known secondary metabolites from both *Hymenoscyphus* species.

## Methods

### Genome and transcriptome sequencing

*Hymenoscyphus fraxineus* (nf4) and *H. albidus* (111/1/4) DNA were extracted and sequenced at the Science for Life laboratories´ (Uppsala Sweden) As described in Stenlid et al. [19] In brief, 500 bp insert libraries were sequenced using a paired end protocol with 100 sequencing cycles from each end on an Illumina HiSeq 2000 sequencer. Long mate pair inserts were sequenced using ABI SOLiD Exact Call Chemistry. For *H. fraxineus*, two libraries with insert sizes of 3 kb and 8 kb were sequenced, and for *H. albidus* a single library with an insert size of 3 kb was sequenced. All mate pair libraries were sequenced with 60 bp from each end.

For transcriptome sequencing, cultures were harvested by filtration and the mycelia were flash frozen in liquid nitrogen and stored at -80 °C until extraction. Total RNA was extracted using RNeasy Plant Mini Kit (Qiagen). Samples were treated with DNaseI (SIGMA) to remove the genomic DNA and the RNA integrity and concentration were measured on a BioAnalyzer 2100 (Agilent). Between 2 and 5 µg of total RNA per sample was sent to Science for Life laboratories (Uppsala, Sweden) for sequencing 100 bp paired end reads on an Illumina HiSeq 2000 platform.

### Quality filtering and assembly

Raw reads from the Illumina platform were quality filtered using Nesoni (https://github.com/Victorian-Bioinformatics-Consortium/nesoni ) to remove low quality sequences and adaptor sequences. Filtering thresholds were set to minimum Q20, and a minimum of 75 bp read length after quality clipping. Only complete pairs were kept for the assembly. Raw reads from the ABI SOLiD platform were quality filtered with the same parameters, except that the minimum read length was set to 35 bp. The genomes were assembled to contigs using Illumina reads with ABYSS version 1.3.6 [49]. K-mer length was optimised to maximize N50. Contigs were subsequently scaffolded using both Illumina paired end reads and the SOLiD long insert reads with SSPACE 2.0 [50] with the additional options to reduce the required overlap (–m 25), minimum coverage to call a base (-o 15) and enable contig overlap extension (-x 1). Finally, gaps were closed using the SOAPdenovo gapcloser utility[51] with default parameters, using Illumina paired end reads. Assemblies were evaluated with FRCAlign [52], and genome completeness was also evaluated using CEGMA [53].

### Gene model annotations

Structural gene annotations were performed with MAKER2 version 2.31.6 [54]. MAKER was configured to use the SNAP, Augustus and GenemarkES [55–57] *ab initio* gene predictors. GenemarkES was trained using the supplied auto-training function. SNAP and Augustus were initially trained using CEGMA annotated genes. Next, proteomes of *Meliniomyces bicolor* strain E, *Neocosmospora* sp. 77-13-4 (= *Nectria haematococca* MPVI) (v2.0), *Botrytis cinerea* B05.10, *Fusarium graminearum* PH-1, and *Neurospora crassa* OR74a were used as evidence in the MAKER runs. Furthermore, we assembled the pure culture RNASeq data into transcripts, using a genome-guided Trinity method [58], which was also provided as evidence to MAKER. After the initial training of SNAP and Augustus, MAKER was run with a single predictor (SNAP or Augustus) to create a first set of hinted- and evidence-supported gene models from the two *ab initio* predictors. These sets were then filtered to only contain genes with RNASeq support, and an annotation edit distance (AED) score less than 0.2. Thereafter, SNAP and Augustus were iteratively retrained using their respective preliminary MAKER gene sets. Finally, MAKER was run with all three predictors enabled, to create a final gene set. Provisional descriptions of the gene models were assigned through the best BLAST hit of the predicted protein against the UniProt database (downloaded 2014-01-31), with an e-value cut-off of 10^− 20^. Automated functional annotations, including e.g. GeneOntology (GO), Pfam domains, signal peptides and transmembrane domains, were assigned to all predicted proteins using InterProScan (version 5.18, database version 57.0). The predictions and evidence were visualised using the JBrowse genome browser (http://jbrowse.org/ ).

To enable comparative genomic analysis of the focal species in a phylogenetic framework, we used other published Helotialean fungi for comparisons. The genomes used were *Ascocoryne sarcoides* NRRL 50,072, *B. cinerea* T4, *Glarea lozoyensis* ATCC 74,030, *Marssonina brunnea* f. sp. *multigermtubi* MB_m1, *Sclerotinia sclerotiorum* 1980, and *Sclerotinia borealis* F-4128, as well as the outgroup *Blumeria graminis* DH14 [59–65]. Since the gene annotations of these species were produced with a variety of different annotation pipelines and vary in age, we re-annotated them with MAKER following the same procedure as we used for the *Hymenoscyphus* species and utilising the available EST or RNASeq evidence that was available in conjunction with the genome sequences.

### Whole genome alignments

Whole genome alignments between *H. fraxineus* and *H. albidus* genomes were established using the whole genome alignment suite Mercator [66]. Mercator needs a set of anchoring points in order to create an initial synteny graph. We used the set of whole genes to establish syntenic blocks, and ran Mercator with default parameters and minimum run length set to 3. Final global alignments of the syntenic blocks were then done using MAVID [67]. *Glarea lozoyensis* ATCC 74,030 [59] was included as an outgroup in the alignments.

### Transposable element analyses

The repeat contents of the genomes were annotated using a combination of RepeatModeler Open-1.0.6 [68] for *de novo* discovery of repeat sequences and RepeatMasker to annotate repeats within the genomic sequence based both on the standard repeat masker libraries and the *de novo* discovered repeat libraries (http://www.repeatmasker.org). All TEs reported at standard settings were used for the subsequent analysis. This includes both complete, but also partial/degraded TEs. Subsequently, three different indexes for the presence of RIP mutations were calculated for each repeat family: RIP product index [69, 70], RIP substrate index [69, 70] and composite RIP index (CRI) [71]. CRI was calculated in non-overlapping 50 bp windows of all TEs and in 500 bp windows of the assemblies with the size larger than 1 Kb. The calculations were performed using the Perl script “RIP_index_calculation.pl” provided by Gioti and co-workers [35]. To quantify the expansion rates of the two largest TE families, LTR/Gypsy and LTR/Copia elements, we first counted the copy numbers for both families in *H. fraxineus*, *H. albidus* and *G. lozoyensis*. Then reciprocal best BLASTN search, using default setings, was performed between the TE sets of the three species. Copies showing any similarity were considered to originate from the common ancestor while the others were considered a result of lineage specific gene expansions. The ratio of total number of copies to that in the ortholog-group was regarded as an estimate of the expansion rate.

### Comparative genomics of *H. fraxineus* and *H. albidus* and tests for selection

One-to-one orthologous between *H. fraxineus* and *H. albidus* genes, and three-way orthologous between *H. fraxineus, H. albidus* and *G. lozoyensis* were established using reciprocal best BLASTP between predicted protein sequences, with a cut off E-value of 1e-20 using NCBI BLAST + 2.2. Transcripts of all gene pairs and triplets were aligned using MACSE version 1.01b [72]. MACSE is a codon-aware aligner, which does the initial alignment on translated amino acid sequences. The alignments were subsequently filtered to exclude any alignment where the ratio between the shortest sequence and total alignment length was below 0.9, to avoid misaligned genes. We used codeml from the PAML package [73] to calculate synonymous divergence, as well as ω values for all gene pairs. To identify genes evolving under positive selection, we again used codeml from the PAML package by comparing codeml model 7 to model 8 using the likelihood ratio test. Briefly, this test compares whether a model allowing three classes of ω ratios (< 1, 1 and > 1) among the sites in the alignments gives a better fit than the model only allowing the two classes of ω ratios < 1 and 1. The obtained P values were corrected for multiple testing using the FDR (Benjamini-Hochberg). To test for overrepresentation of functional groups (defined as GO categories) among the non-neutrally evolving genes we compared the frequencies of GO categories within the group versus the frequency in the complete proteome. We used Fisher´s exact test, with the FDR applied to correct for multiple testing, as implemented in goatools[74].

### Identification of gene families and evolution

Gene families were established using OrthoMCL [75]. Protein sets from our annotations of the Helotialean species were clustered with an expansion factor parameter set to 1.5. To test for expansions and contractions among the gene families, we used CAFE version 3.1 [76] with default settings and let the program estimate the underlying birth-death ratio. CAFE requires an ultrametric phylogenetic tree describing the relationship between the species. This tree was constructed as described in [19].

### Annotation of secondary metabolite biosynthesis gene clusters (BGCs)

BGCs were annotated in all genomes using the antiSMASH pipeline version 3.0.5.1 run in stand-alone mode [77]. Conservation of BGCs between *Hymenoscyphus* spp. and *G. lozoyensis* was evaluated by ortholog sharing. Any two BGCs sharing three or more orthologous genes between the species, as identified by OrthoMCL clusters, were considered shared between the species.

### Annotation of specific BGCs in *H. fraxineus* and *H. albidus* genomes

In order to identify the BGCs of viridiol and hymenosetin in our examined *Hymenoscyphus* genomes, we used previously described BGCs of fungal metabolites with high structure similarity to these two known secondary metabolites [39, 43, 78–80]. The *H. fraxineus* and *H. albidus* protein databases were queried with protein sequences of putatively related BGCs using the BLASTP algorithm to identify high scoring sequence alignments.

In the case of viridiol, our analyses were based on the *vid* cluster responsible for the assembly of the viridiol congener demethoxyviridin that was found in a *Nodulisporium* species [78, 81]. The Baeyer-Villiger monooxygenase VidF (Genbank ID AVY05513) of the *vid* cluster served as template for the manual BLASTP search against the two *Hymenoscyphus* protein databases. Thereafter, a synteny analysis was conducted between the *Nodulisporium vid* BGC and the candidate cluster from *H. fraxineus* and *H. albidus* to confirm their potential role as viridiol BGC. Similarity searches were based on the TBLASTX algorithm and visualized with the clinker tool[82]. Likewise, for the localization of the hymenosetin BGC we used the known equisetin (*eqx*) BGC from *Fusarium heterosporum* [79, 80]. Equisetin is a 3-decalinoyltetramic acid derivative with high structure similarity to hymenosetin [43]. BLASTP search for high scoring sequence alignments with the protein sequences of the 11 members of the *eqx* BGC in the two *Hymenoscyphus* genomes was performed followed by a synteny analysis of the results to reveal a potential candidate gene cluster for hymenosetin.

## Results

### High genome similarity in *Hymenoscyphus* synteny blocks

The genome assembly of *H. fraxineus* comprises 137 scaffolds with an N50 of 997 kb for a total sequence length of 64.2 Mb. The genome assembly of *H. albidus* comprises 756 scaffolds with an N50 of 135 kb for a total sequence length of 51.2 Mb (Table 1). Both genomes are AT-rich, with a G + C content of 37.4 and 44.6 % in *H. fraxineus* and *H. albidus*, respectively.

**Table 1:** Summary statistics for the assemblies of *Hymenoscyphus fraxineus* and *H. albidus*

	*H. fraxineus*	*H. albidus*
Assembly length:	64.2 Mb	51.2 Mb
Gap length:	0.45 Mb (0.71 %)	0.42 Mb (0.83 %)
G + C content:	40.10 %	44.40 %
Longest scaffold:	2.99 Mb	0.61 Mb
N50:	997 kb (n:20)	135 kb (n:108)
Number of scaffolds > 1 kb	137	756
CEGMA Complete	96.4 %	95.6 %
CEGMA Partial	97.2 %	96.8 %

**Fig. 1:**
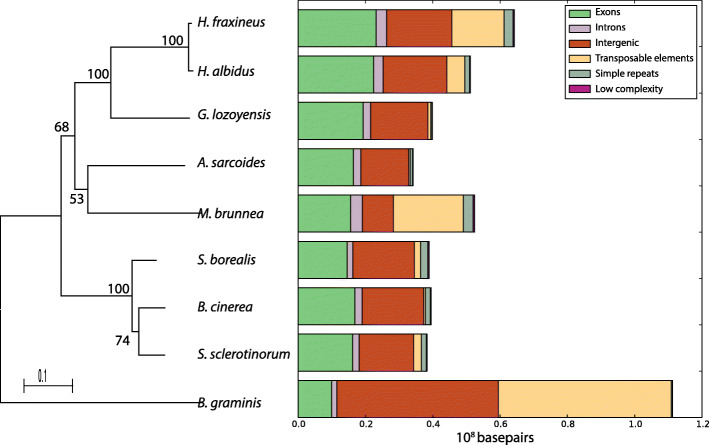
Genome composition of ***H. fraxineus***, ***H. albidus*** and seven other Helotialean species. Gene models and repeats were predicted using the same MAKER and RepeatMasker pipelines, for all eight fungal genomes in the Helotiales (*Hymenoscyphus fraxineus, Hymenoscypus albidus, Glarea lozoyensis, Ascocoryne sarcoides, Marssonina brunnea, Sclerotinia borealis, Sclerotinia sclerotiorum* and *Botrytis cinerea)* and the outgroup *Blumeria graminis*

Gene models were predicted using the same MAKER pipeline for all eight fungal genomes in the Helotiales and the outgroup *B. graminis*, in order to minimise any biases due to different gene prediction methodologies. In total 14,069 and 13,947 putative genes were identified in *H. fraxineus* and *H. albidus*, respectively (Supplementary table S1). The coding gene space occupies approximately 25 Mb in both *Hymenoscyphus* species (Fig. 1), which is the largest among all compared Helotiales genomes: only *G. lozoyensis* has an approximate coding gene space larger than 20 Mb. 53 and 54 % of the predicted transcripts in *H. fraxineus* and *H. albidus*, respectively, were supported by gene expression data. However, on average only 58 % of the genes were assigned to Pfam domains and 39 % had UniProt homologies, which were lower than the six other Helotialean species (Pfam: from 61 to 65 %; UniProt: from 42 to 48 %, Supplementary table S1).

A whole genome alignment of *H. fraxineus* and *H. albidus* was produced using the Mercator whole genome alignment suite. Mercator alignments were anchored using whole genes, thus providing synteny blocks of genes between the genomes. We found 10,427 syntenic blocks between the two *Hymenoscyphus* species with the largest block of 105 genes and a block N50 of 21 genes (i.e. 50 % of the genes are found in syntenic blocks consisting of 21 genes or more).

We also evaluated DNA sequence conservation in 1 kb sliding windows along the genomes. Comparing *H. fraxineus* and *H. albidus*, we found 39.1 Mb sequences aligned with an average of genome-wide sequence identity equal to 77 %.

### Recent expansion of transposable elements in *Hymenoscyphus*

Most of the genome size differences between the two *Hymenoscyphus* species can be attributed to increased dispersed repeat content in *H. fraxineus*. In general, *H. fraxineus* had 24.2 % of its genome assembly identified as TEs while this number was 10.4 % in *H. albidus* (Fig. 1, and Table 2). The observed difference in repeat content was not only caused by copy number differences (3410 more repeats are detected in *H. fraxineus* than in *H. albidus*) but also due to the fact that they were on average 2.9 times longer in *H. fraxineus* than those of *H. albidus* (Supplementary table S2). For example, a markedly larger number of DNA/TcMar-Fot1 copies were identified in *H. fraxineus* (1335 versus 7 in *H. albidus*), the TE copies also had a significantly longer mean length (479 bp compared to 106 bp). Additionally, large parts of the repeat contents (7 Mb of the *H. fraxineus* and 3 Mb of the *H. albidus* genomes) were potentially novel and identified as “unknown” by RepeatModeler. In contrast to *Hymenoscyphus* spp., the percentages of the dispersed repeat contents in *G. lozoyensis* were low (2.2 %, Fig. 1; Table 2). However, it is well known that TE-rich genomes can be problematic to assemble with short-read technologies. The observed size difference between the assemblies is 11 Mb. Moreover, the expected size difference between the genomes can be extrapolated from Illumina read coverage on the 50 % largest scaffolds. The extrapolation indicates that the size difference between the genomes is 4 Mb. This suggests that while some of the difference might be explained by collapsed repeats in the *H. albidus* assembly at least 4 Mb of size difference can be attributed to true differences between the genomes.

**Table 2:** The distributions of TEs, RIP, CRI, and GC percentages

Species	TEs%^a^	RIP% (genome)^b^	RIP% (TE)^c^	mean CRI (RIP)^d^	GC% (genome)^e^	GC% (TE)^f^	GC% ratio^g^
*H. fraxineus*	24.16	40.85	92.91	1.66	37.37	33.87	0.59
* H. albidus*	10.44	24.28	58.47	1.15	44.58	41.59	0.87
*G. lozoyensis*	2.17	9.47	87.77	1.75	45.74	35.7	0.75
* A. sarcoides*	1.48	11.0	79.63	1.25	46.49	41.49	0.48
*M. brunnea*	39.95	46.31	76.80	1.08	40.99	36.15	0.88
* S. borealis*	4.66	8.05	40.97	0.63	39.40	35.50	1.06
*B. cinerea*	1.17	2.87	38.19	0.7	43.07	38.05	1.00
* S. sclerotiorum*	5.88	6.42	39.00	0.75	41.78	39.10	1.00
*B. graminis*	43.24	6.98	27.93	0.67	41.88	43.69	0.96

^a^ the percentage of dispersed repeats (transposable elements) in the genomes; ^b^ the percentage of detected RIP signatures across the whole genome; ^c^ the percentage of detected RIP signatures across the TE’s in the genome; ^d^ mean composite RIP index; ^e^ the percentage of GC nucletides in the whole genome; ^f^ the percentage of GC nucleotides in the TE’s and ^g^: mean GC% ratio of RIPed to unRIPed TEs across all types

To identify the fraction of RIP-inactivated TEs, we calculated a Composite RIP index, CRI, in sliding windows of the whole genome as well as of TEs. On average, 40.9 % of the *H. fraxineus* genome showed characteristics of RIP (CRI > 0) while only 24.3 % of the *H. albidus* genome had such signatures. Altogether, 92.9 % of TE sequences in *H. fraxineus* were inactivated by RIP (mean CRI = 1.66) whereas only 58.5 % of TEs in *H. albidus* were inactivated by RIP (mean CRI = 1.15) (Table 2). RIP inactivated TEs are accompanied by lower GC content [71]. *Hymenoscyphus albidus* had a higher GC% compared to *H. fraxineus* across the whole genome (44.6 % versus 37.4 %) as well as in TEs (41.6 % versus 33.9 %). The average ratio of GC% comparing TEs with RIP, to TEs without RIP was 0.87 in *H. albidus*, higher than that in *H. fraxineus* (0.59). In summary, 16 TE families in *H. fraxineus* were identified to have typical RIP patterns, including large sizes of RIP regions, high CRI values, and low GC%, especially in Long-terminal repeat (LTR)/Copia and LTR/Gypsy elements (Supplementary table S3). In contrast, only three TE families in *H. albidus* were enriched for such RIP patterns.

We further tried to quantify the copy number expansion rates of LTR/Gypsy and LTR/Copia elements, the two largest TE families in *H. fraxineus*, *H. albidus* and *G. lozoyensis* since their most recent common ancestor. For LTR/Gypsy, in total 1866, 1915, and 405 copies were identified in the three species, respectively. The LTR/Gypsy elements were on average 2.6 times longer in *H. fraxineus* than in *H. albidus* and 2.9 times longer than in *G. lozoyensis* (Supplementary Table 2). Only 78, 4, and 17 copies, respectively, were detected by reciprocal BLASTN analysis between the TE sets. If we make the conservative assumption of an ancestral copy number of at most 78 we estimate approximately a 24-fold increase in *H. fraxineus* and *H. albidus* LTR/Gypsy, whereas a five-fold LTR/Gypsy expansion was estimated for *G. lozoyensis.* In *H. albidus* RIP signatures were largely absent in this TE family in contrast to *H. fraxineus* and *G. lozoyensis* (Supplementary table S3). On the other hand, for LTR/Copia all three species showed enriched RIP signatures (Supplementary table S3). Among these repeats we found 3320, 491, and 64 copies for *H. fraxineus*, *H. albidus* and *G. lozoyensis*, respectively. Again the TE’s were longer in H. fraxineus than in the other two taxa (Supplementary table S2). BLASTN similarity analysis identified 715 conserved copies for *H. fraxineus* and 164 copies for *H. albidus* but none for *G. lozoyensis*. This case is consistent to the genomic background *H. fraxineus* showing higher expansion rate than *H. albidus* (4.6 versus 3-fold, fisher exact test *p*-value = 5.94e-9).

### Relatively few genes show signs of positive selection between *H. fraxineus and H. albidus*

To test for signs of positive selection, we created two- and three-way alignments of genes found to be two-way orthologs between the two *Hymenoscyphus* species, or three-way orthologues between *H. fraxineus, H. albidus* and *G. lozoyensis.*

**Fig. 2:**
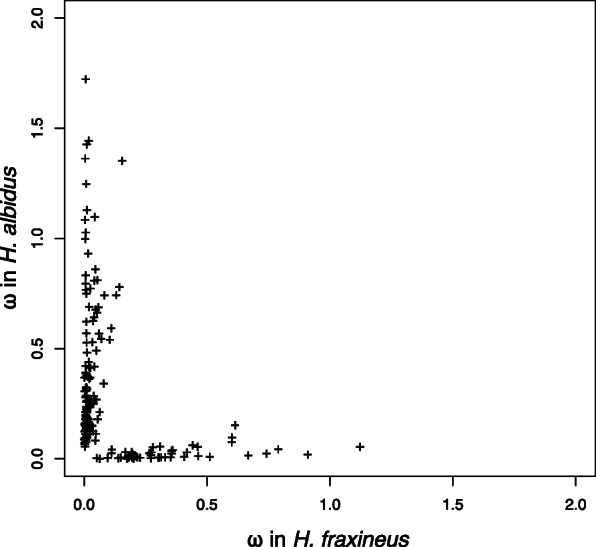
Plot of ω values in genes differently evolving in ***H. fraxineus*** (outcrossing) and ***H. albidus*** (selfing). In this case differently evolving means better fit of our data to a two-ratio model allowing for a difference in ω between *H. fraxineus* and *H. albidus* branches in the tree. For clarity only the differentially evolving genes are plotted

In the pairwise comparisons, we used a maximum likelihood approach as implemented in the PAML package to test a model allowing for sites with positive selection, neutral evolution, and negative selection (ratios of synonymous to non-synonymous changes in the DNA sequence, ω > 1, ω = 1, and ω < 1 respectively) versus a model only allowing for negative selection or neutral evolution (ω < 1 and ω = 1). We identified 186 genes showing signs of positive selection (likelihood ratio test, p < 0.05 after Holm correction for multiple testing) (Fig. 2, Supplementary table S4). Gene Ontology annotations on the gene set indicates that most genes detected are involved in primary metabolism. One notable exception is a significant enrichment of GO:0044425 (membrane_part), indicating a membrane localisation of these proteins. In the three-way comparison, we instead tested a model assuming one ω shared between the two *Hymenoscyphus* species, versus a model where each species was allowed to have a separate ω, thus effectively testing for genes where the function has diverged between the two *Hymenoscyphus* species. We identified 59 genes where a model with different ω for the *Hymenoscyphus* species had a better fit than with constant ω (Supplementary table S4). Among these genes, 34 had UniProt homologies.

### Secondary metabolite gene families in *Hymenoscyphus* show signs of rapid changes

An analysis of the evolution of the sizes of OrthoMCL gene families among the Helotiales genomes using CAFE identified 26 gene families that showed significant changes in copy numbers of paralogous genes between the two *Hymenoscyphus* species or on the branch to *Hymenoscyphus/ Glarea* (Table 3). Eight of these gene families (omcl1001, omcl1016, omcl1018, omcl1241, omcl1242, omcl1262, omcl1371 and omcl1531) were associated with specialized metabolite synthesis or transport (Table 3). The family omcl1241, with four members in *H. fraxineus* and one member in the other two species, comprise homologs of the *Versicolorin B synthase* (*vbs*) genes (Table 3). Similarly, omcl1371 has four members in *H. fraxineus* compared to one member in *H. albidus* and two in *G. lozoyensis*. Two of the gene families expanded in the ancestor of *Hymenoscyphus/ Glarea*, omcl1001 and omcl1531, encode homologs to enzymes catalysing key steps in the lovastatin polyketide biosynthesis pathway. A small number of genes associated with these expanding/contracting gene families showed signs of functional divergence between the two *Hymenoscyphus* species, i.e. the HYFRA_T00003353_1/ HYALB_T00010143_1, HYFRA_T00002327_1/HYALB_T00002704_1 (both members of omcl1001) and the HYFRA_T00000332_1/HYALB_T00012128_1 (omcl1018) gene pairs (Supplementary table S4), indicating a divergence of function of these gene pairs in the two species. It should be noted that another group of gene families that showed signs of rapid changes in numbers of paralogous genes were families comprising *Vegetative incompatibility protein / HET homolog* genes (omcl1014, omcl1037 and omcl1185, Table 3).

**Table 3:** OMCL gene families showing rapid changes in numbers of paralogs in *Hymenoscyphus*

Gene Familiy	*H. fraxineus*	*H. albidus*	*G. lozoyensis*	Median of other	Description
**Specific expansion in* H. fraxineus***					
omcl1208	3	2	0	1	Multicopper oxidase / Laccase
omcl1241	4	1	1	1	Versicolorin B synthase homologs
omcl1267	5	2	2	1	PhospholipaseD like domain containing proteins (PFAM 13,091)
omcl1371	4	1	2	1	Major Facilitator Superfamily transporters
omcl1663	10	0	0	0	Protein of unknown function
**Specific expansion in* H. albidus***					
omcl1014	1	10	3	2	Vegetative incompatibility protein / HET homologs
omcl1166	0	16	0	0	Protein of unknown function
omcl1662	0	10	0	0	Protein of unknown function
omcl1037	2	6	2	1	Vegetative incompatibility protein / HET homologs
omcl1431	0	3	1	0	Ankyrin repeat containing proteins
omcl1473	1	3	0	0	Protein of unknown function
omcl1564	0	3	1	1	Protein of unknown function
omcl1661	0	1	0	0	Protein of unknown function
**Expansion in ancestral species**					
omcl1001	22	23	23	7	NRPS/PKS, LovB homologs
omcl1004	9	9	13	6	Glycosyl hydrolase family 3
omcl1016	5	8	8	3	ABC transporters
omcl1018	6	5	7	2	NRPS
omcl1185	2	2	5	1	Vegetative incompatibility protein / HET homologs
omcl1204	1	2	9	0	Protein of unknown function
omcl1215	3	2	2	1	Contains pyridine nucleotide-disulphide oxidoreductase PFAM domain
omcl1242	2	1	3	0	PKS
omcl1262	2	3	3	0	NACHT domain containing proteins
omcl1311	3	2	3	0	Protein of unknown function
omcl1322	2	1	3	1	Protein of unknown function
omcl1385	3	2	2	0	Protein of unknown function
omcl1417	1	3	2	1	Protein of unknown function
omcl1444	2	1	2	1	Protein of unknown function
omcl1531	2	1	3	0	enoyl reductase, LovC homologs

### Identification of the viridiol and hymenosetin BGCs in *H. fraxineus* and *H. albidus*

Both *H. albidus* and *H. fraxineus* are known to produce specialized metabolites that are harmful to their host under laboratory conditions. On a general term, both species have a large specialized biosynthetic gene cluster (BGC) repertoire, and there were prominent overlaps in the repertoire between *H. albidus* and *H. fraxineus* as predicted by antiSMASH (Supplementary figure S1).

**Fig. 3:**
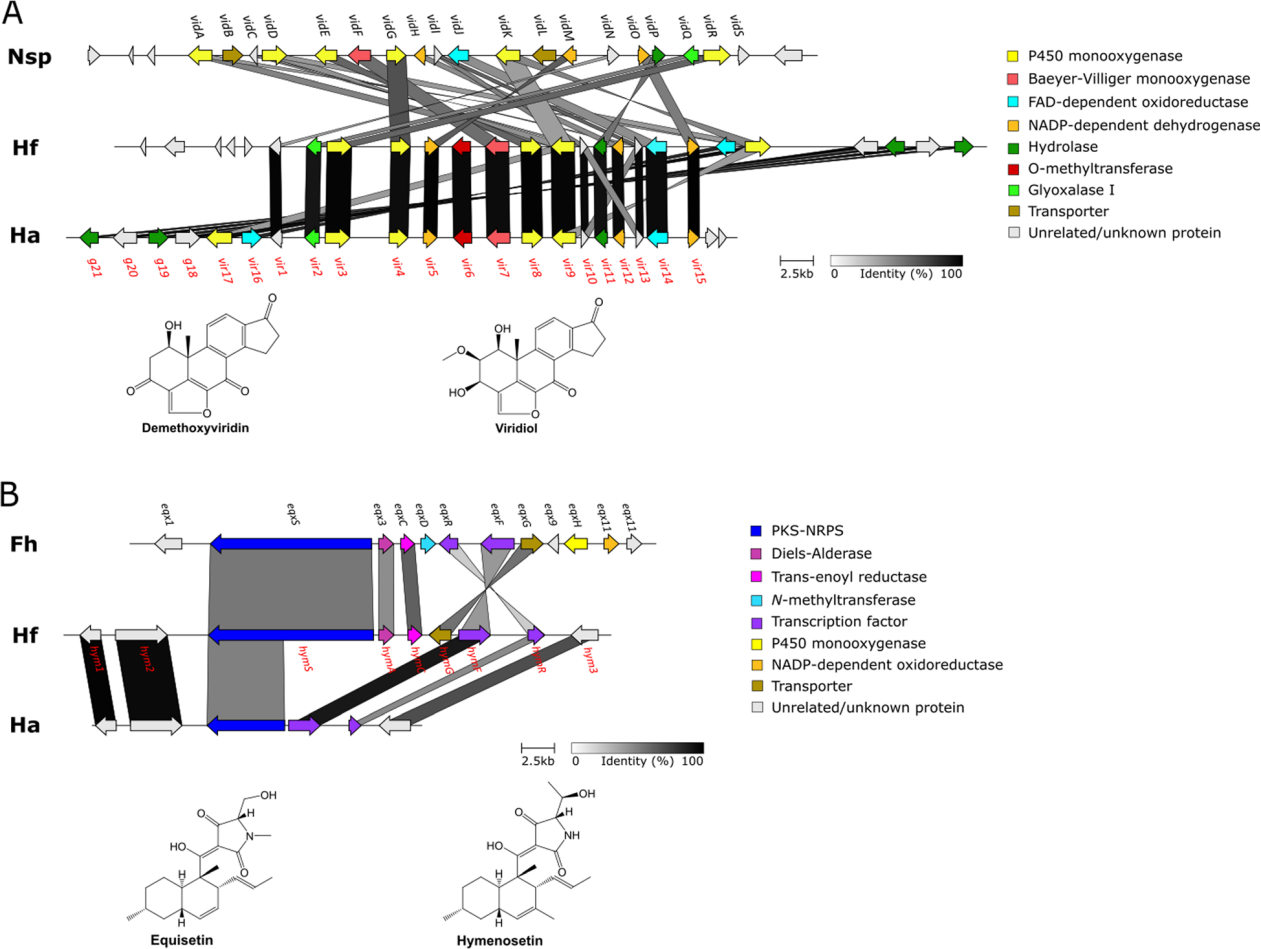
Biosynthetic gene cluster (BGC) comparison of ***H. fraxineus*** and ***H. albidus*** BGCs associated with the production of viridiol and hymenosetin. The clusters were identified using TBLASTX visualized by clinker: (**a**) Gene map of the known demethoxyviridin (*vid*) BGC from *Nodulisporium* sp. (*Nsp*) and the identified homologous viridiol (*vir*) BGC from *H. fraxineus* nf4 (*Hf*) and *H. albidus* 111/1/4 (*Ha*). The structures of the respective pathway products are shown; (**b**) Gene map of the known equisetin (*eqx*) BGC from *Fusarium heterosporum* (*Fh*) and the respective homologous *hym* BGC from *H. fraxineus* nf4 (*Hf*) putatively responsible for hymenosetin production. In *H. albidus*, most likely a deletion event of a 10 kb fragment of genomic DNA took place within the *hym* cluster and lead to the absence of essential members of the *hym* cluster. The structures of the respective pathway products are shown below

No likely viridiol BGC could be localized with the antiSMASH search algorithm. Assuming the viridiol cluster in *Hymenoscyphus* is organized similar to the *vid* cluster in *Nodulisporium spp.*, the cluster would not encode for a core gene and can thus not be detected by antiSMASH. The manual BLASTP search using the Baeyer-Villiger monooxygenase VidF returned high scoring sequence alignments in both *Hymenoscyphus* species (Fig. 3 A, Supplementary table S5). The best hit, named *vir7*, in both *Hymenoscyphus* species showed a 64.4 % identity to VidF and was located in a cluster containing several P450 monoxygenases.

The synteny analysis demonstrated that both *Hymenoscyphus* candidate clusters contain homologs of most genes of the *Nodulisporium vid* cluster (Fig. 3 A, Supplementary table S5), indicating that they are indeed representing the putative viridiol (*vir*) BGC. Compared to the *vid* cluster, both *vir* clusters in *Hymenoscyphus* have gained an additional *O*-methyltransferase (*vir6*) and a FAD-dependent oxidoreductase (*vir16*) (Fig. 3 A). The synteny analysis further showed that the *vir* cluster is, for the most parts, conserved between the two *Hymenoscyphus* species. Interestingly, *vir3* genes in *H. fraxineus* and *H. albidus* (HYFRA_T00008109_1 and HYALB_T00010878_1) are among those genes that showed signs of positive selection, or relaxed purifying selection, in the two species comparison (Supplementary table S4).

A similar query of the *H. fraxineus* and *H. albidus* protein catalogues with protein sequences from the *Fusarium heterosporum* equisetin *(eqx)* gene cluster yielded high scoring sequence alignments for the *eqx* gene cluster proteins EqxS, Eqx3, EqxC, EqxR, EqxF and EqxGin *H. fraxineus*, while only EqxR, EqxF and partly EqxS produced significant hits in the predicted *H. albidus* proteome. The six significant hits in *H. fraxineus* were located consecutively on scaffold 52 in the genome forming the putative hymenosetin, *hym*, gene cluster (Fig. 3B, Supplementary table S6). The antiSMASH scan for BGCs identified the *hymS, hymC* and *hymG* in the putative hymenosetin gene cluster in *H. fraxineus* genome but returned no hits for *H. albidus*. The *hymS* gene belongs to the omcl1001 gene family which is expanded on the branch to *Hymenoscyphus/ Glarea*, while the *hymG* gene belongs to the omcl1371 gene family which is expanded in *H. fraxineus* specifically (Table 3, Supplementary table S6). The subsequent synteny analysis of the putative *hym* BGC with the *eqx* BGC in *F. heterosporum* showed that homologs of *eqxD* (a *N*-methyltransferase), *eqxH* (a P450 monooxygenase) and *eqx11* (a NADP-dependent oxidoreductase) were missing (Fig. 3B). However, neither *eqxH* nor *eqx11* are involved in the biosynthesis of equisetin [79, 80].

## Discussion

### *H. albidus* may be less well equipped to adapt to a rapidly changing environment than *H. fraxineus*

The high synteny of the *Hymenoscyphus* genomes, absence of large genome rearrangements (this study) and high similarity in e.g. CAZYme profiles as reported by Stenlid and co-authors [19] indicates that more subtle differences in gene content and expression contribute to the observed difference in lifestyle between the two species on European ash. TE activity can contribute to faster evolution of virulence traits [20, 21], and in fact, we observed almost twice as many TEs in *H. fraxineus* as in *H. albidus*. This observation is in line with the reports that some, but not all, filamentous pathogens have high proportion of TEs and repeat-rich regions in their genome; features which suggest faster evolution of virulence traits [21, 83]. We acknowledge that the assembly of *H. albidus* is more fragmented than the *H. fraxineus* assembly. As repeat-sequences are hard to assemble with short reads. This could potentially contribute to the differences we observe between the two genomes. However, the patterns are not uniform for all TEs, there are several TE families which appear to be more numerous as well as longer in *H. albidus.* This suggests that assembly quality is likely not the sole driver of the patterns we observe. Furthermore, we observed approximately twice as many RIPs in the *H. fraxineus* genome than in the genome of *H. albidus*, as well as a markedly lower GC content ratio within TE regions compared to the background genome in the former species. As RIP tends to produce C to T mutations it could thus be regarded as a relic of RIPs [33, 71]. Taken together, the data suggested that the branch of *Hymenoscyphus* had an TE invasion after it split from *Glarea*, which greatly elevated TE percentages in the genome from 1.5 ~ 2 to at least ~ 10 %. Thereafter, a second TE expansion may have occurred specifically on the branch leading to *H. fraxineus*.

The TE and RIP patters can also be considered against the backdrop of the mating systems in *H. albidus* and *H. fraxineus* [18] as changes in trophic strategies and/or in breeding systems may have a vast impact on genome evolution, e.g. accumulation of deleterious mutations in selfing species [84]. It is often thought that transitions from outcrossing to self-fertility would lead to an evolutionary dead-end, i.e. eventually the extinction of species [25, 85]. The dead-end hypothesis has been examined in the genus *Neurospora* [35]. In this genus, different signatures were observed in different TE families. In the most abundant TE family Gypsy, lower copy numbers and reduced RIP frequencies were observed in selfing taxa of *Neurospora* which was interpreted as a burst of Gypsy family TEs specific to the outcrossing species [35]. In *Hymenoscyphus*, the two species had approximately similar copy numbers of Gypsy, but those in *H. fraxineus* were on average much longer, and signals of RIP were also only observed in *H. fraxineus*. An increase of the TE content has been associated with a pathogenic lifestyle [24, 36]. However, given that both *Hymenoscyphus* species appear to have extensive saprotrophic capacities and produce phytotoxic substances [17, 19, 44, 48] it seems unlikely that expansion of TEs is associated with a change of lifestyle from saprotrophism to pathogenicity. It is possible that the TE expansion is simply the result of differences in mating system since genomes of outcrossing taxa tend to be capable to bear higher TE load (higher efficiency of purifying selection) or have higher efficiency of TE silencing by RIP compared to selfing species [35]. Because transition to selfing is usually regarded as unidirectional and irreversible [86], we thus extrapolate that the TE expansion within the *Hymenoscyphus* branch stopped or slowed down greatly in *H. albidus* after the mating system transition but still continued in *H. fraxineus*.

Gioti et al. [35] pointed out that *Neurospora* selfing species accumulate deleterious alleles (elevated ω) that could be evidence for a relaxation of purifying selection in protein-coding genes. In selfing species, an increased hitch-hiking effect and reduced effective population size would both reduce the effect of purifying selection and lead to accumulation of deleterious alleles [87, 88]. In our case, only a very limited number of genes showed significant signals of positive selection (i.e. high ω), yet more genes with signs of elevated ω were detected in the selfing species *H. albidus*, possibly indicating that its purifying selection is less efficacious compared to *H. fraxineus.* Purifying selection together with genetic drift are the major forces that drive divergence between genomes of outcrossing and selfing species, including among transposable elements [30, 85, 89]. The latter is also observed in the shorter mean lengths of TEs in *H. albidus*, indicating that TE have been eroded at a higher rate in *H. albidus*.

Taken together, the results from our comparison of the two *Hymenoscyphus* species genomes can be interpreted as concordant with the dead-end hypothesis [35, 85], where the lack of adaptive potential in the selfing species, *H. albidus*, leaves it poorly equipped to adapt to a rapidly changing environment; in this case the invasion of the out-crossing congener *H. fraxineus* that has assumed the same ecological niche and competitively excluded *H. albidus* [90], and as a result drastically reduced the population size of their host *F. excelsior*[4].

### Signs of potential adaptive differentiation in the specialized metabolite repertoire in *Hymenoscyphus*

The fungal genomes in this study had high similarity in their compositions of both genic and intergenic regions, especially when comparing the two *Hymenoscyphus* species (*H. fraxineus* and *H. albidus*). However, focused analyses of the *H. fraxineus* and *H. albidus* specialized metabolite biosynthesis genes and BGC repertoire indicate putatively adaptive changes in the fungi, that could be a result of their adaption to different hosts or, less likely, different trophic strategies [43, 91]. Although both *Hymenoscyphus* species have relatively large BGC repertoires, the *H. fraxineus* genome appear to possess a broader specialized metabolite arsenal than its congener. The broad BGC repertoire in *Hymenoscyphus* is consistent with the idea that specialized metabolites or metabolite functions may provide an adaptive advantage to fungi interacting with other organisms, and with the *Hymenoscyphus* species niche in leaf litter, where they may need to protect their substrate, the degrading rachises’, through antibiosis [17, 22, 38]. It has been reported that the genome composition of the relatively closely related species *G. lozoyensis* also indicates a large potential to produce a diverse array of specialized metabolites, in comparison to *A. sarcoides* [59, 92]. Our analysis of the BGC repertoires and gene family expansions agrees with these reports, suggesting the possibility that the lineage leading to *Hymenoscyphus/Glarea* accumulated duplications and neofunctionalisations of BGCs over a relatively long evolutionary time frame. However, when specifically comparing the two *Hymenoscyphus* species, *H. fraxineus* appeared to have a slightly broader specialized metabolite arsenal than *H. albidus* based on the detected gene family expansions/contractions and BGC repertoires. To understand the role of these, possibly adaptive, differences in the genome content for the ecology of *H. fraxineus* or *H. albidus*, detailed analyses of the BGCs are needed. This is perhaps best illustrated by the analysis of the viridiol, *vir*, and hymenosetin, *hym*, gene clusters.

A putative BGC for viridiol is present in each of the examined *Hymenoscyphus* species. The identification of the first complete viridiol (*vir*) BGC now allows the full reconstitution of the biosynthetic pathway of viridiol and its analogues known from *H. fraxineus* [39, 40, 42]. While the *vir* cluster structure has been completely reorganized compared to the *Nodulisporium* sp. *vid* cluster, the *vir* BGCs were for the most part conserved between the two *Hymenoscyphus* species. Besides, the synteny appeared to extend beyond the viridiol (*vir*) BGC as indicated by the presence of other highly similar genes (i.a. hydrolases) in the proximity of the *vir* cluster in both producer organisms. Apparent divergence between the *vid* cluster and both *vir* clusters can be linked to observed structural differences between demethoxyviridin and viridiol [78, 93]. Particularly, the presence of *vir6* (an *O*-methyltransferase) and *vir16* (a FAD-dependent oxidoreductase) in the *vir* BGC correlates well with the presence of a methoxy functionality in viridiol. Thus, the lack of these genes in *Nodulisporium* sp. (now *Hypoxylon* cf. *croceum* [81]) may explain why this fungus is not able to synthesize viridiol [78]. Taken together, the identification and analysis of the viridiol BGC in *H. fraxineus* and *H. albidus* further suggests that viridiol is not involved in the pathogenicity of *H. fraxineus* and that the *vir* cluster has already appeared before the two fungi had diverged.

A specialized metabolite that may provide *H. fraxineus* with an advantage when it comes to substrate capture *in planta*, is hymenosetin. This metabolite is reported to be secreted by virulent cultures of *H. fraxineus* and shows broad-spectrum microbial activities [43]. In this study, we identified the putative hymenosetin (*hym*) BGC *in H. fraxineus* based on the known equisetin (*eqx*) BGC from *Fusarium heterosporum* [79, 80]. Unlike the *vir* BGC, which could be identified in both *Hymenoscyphus* species, only a part of the central polyketide synthase-nonribosomal peptide synthetase (PKS-NRPS) of the *hym* BGC, *hymS*, as well as the transcription factors *hymF* and *hymR* and three potentially unrelated genes (*hym1*, *hym2* and *hym3*) were identified in the *H. albidus* genome. This observation together with the fact that *H. albidus* never have been shown to produce hymenosetin despite extensive screening efforts (S. Halecker, data not shown), suggest that the *hym* cluster, and capacity to produce hymenosetin, may have has been lost in this species. Presumably, a deletion event of a 10 kb stretch of genomic DNA within the cluster lead to the absence of essential members of the *hym* cluster in *H. albidus*.

In *H. fraxineus*, the *hym* BGC comprised the PKS-NRPS *hymS*, and genes encoding trans-enoyl reductase (*hymC*), a putative Diels-Alderase (*hymA*), a transporter (*hymG*) und two transcription factors (*hymF*, *hymR*). In contrast to the *eqx* cluster, a *N*-methyltransferase, a P450 monooxygenase and a NADP-dependent oxidoreductase gene were missing. The latter two of these have been demonstrated to be not involved in the biosynthesis of equisetin [79, 80] and thus it is likely that they would not be needed for hymenosetin biosynthesis either. The absence of the N-methyltransferase gene in the *hym* BGC correlates with an unmethylated nitrogen in the L-threonine of hymenosetin [43]. The additional methyl group in the decalin scaffold of hymenosetin [43] is possibly the result of different catalytic properties of the methyltransferase domain of the PKS-NRPS core enzyme, while the conjugation of the polyketide unit to L-threonine instead of L-serine is putatively based on the substrate selectivity of the adenylation (A) domain. Therefore, the identified candidate cluster *hym* would fully account for the biosynthesis of hymenosetin in *H. fraxineus*.

As mentioned above, BGCs and the production of specialized metabolites are often tightly associated with ecological niche adaptation of fungi [22, 38] and thus the BGC repertoire and composition are variable among species. The likely loss of the *hym* BGC in *H. albidus* and the findings that genes in BGCs either are members of gene families showing signs of rapid expansions/contractions (*hymS* and *hymG*) or are evolving non-neutrally (*vir3*) are in line with the observations that BGCs frequently show signs of diversification [38, 94], suggesting some ongoing selection for changes in the metabolome of *Hymenoscyphus*.

In conclusion, the genomes of *H. fraxineus* and *H. albidus* show a high degree of synteny, but the results from our analyses of the two species genomes indicate: First, that the species have evolved somewhat different specialized metabolite profiles since their divergence; and secondly, in accordance with the dead-end hypothesis, that the genomic signatures in *H. albidus* may suggest that its transition to homothallism (selfing) made it poorly equipped to adapt to maintain its ecological niche in a rapidly changing environment and the invasion of the out-crossing congener *H. fraxineus.*

## Supplementary Information

**Additional file 1:**

**Additional file 2:**

**Additional file 3:**

**Additional file 4:**

**Additional file 5:**

**Additional file 6:**

**Additional file 7:**

## Data Availability

Assemblies, annotations and short read data have been deposited into the International Nucleotides Sequence Database / ENA-EMBL under accessions PRJEB14441 and PRJEB14442 for *H. fraxineus* and *H. albidus*, respectively. Re-annotations of the Helotialean species, functional annotations of all studied species, as well as predicted transcript and protein sequences are available in the Zenodo repository under DOI: 10.5281/zenodo.4355824. Further requests for materials should be addressed to M.B.D. (mikael.durling@slu.se).

## References

[1] Santini A, Ghelardini L, De Pace C, Desprez-Loustau ML, Capretti P, Chandelier A, Cech T, Chira D, Diamandis S, Gaitniekis T (2013). Biogeographical patterns and determinants of invasion by forest pathogens in Europe. New Phytologist.

[2] Przybyl K (2002). Fungi associated with necrotic apical parts of *Fraxinus excelsior* shoots. Forest Pathology.

[3] Juodvalkis A, Vasiliauskas A (2002). Drying extent of Lithuanian ash-tree woods and factors predetermining it. Vagos.

[4] Enderle R, Stenlid J, Vasaitis R: An overview of ash (*Fraxinus* spp.) and the ash dieback disease in Europe. CAB Reviews 2019, 14:No. 025.

[5] Baral H-O, Queloz V, Hosoya T (2014). *Hymenoscyphus fraxineus*, the correct scientific name for the fungus causing ash dieback in Europe. IMA fungus.

[6] Han J-G, Shrestha B, Hosoya T, Lee K-H, Sung G-H, Shin H-D (2014). First Report of the Ash Dieback Pathogen *Hymenoscyphus fraxineus* in Korea. Mycobiology.

[7] Hosoya T, Otani Y, Furuya K (1993). Materials for the fungus flora of Japan (46). Transactions of the Mycological Society of Japan.

[8] Zhao Y-J, Hosoya T, Baral H-O, Hosaka K, Kakishima M (2012). *Hymenoscyphus pseudoalbidus*, the correct name for *Lambertella albida* reported from Japan. Mycotaxon.

[9] Zheng H-D, Zhuang W-Y (2014). *Hymenoscyphus albidoides* sp. nov. and *H. pseudoalbidus* from China. Mycological Progress.

[10] Cleary M, Nguyen D, Marciulyniene D, Berlin A, Vasaitis R, Stenlid J (2016). Friend or foe? Biological and ecological traits of the European ash dieback pathogen *Hymenoscyphus fraxineus* in its native environment. Scientific Reports.

[11] Gross A, Holdenrieder O, Pautasso M, Queloz V, Sieber TN (2014). *Hymenoscyphus pseudoalbidus*, the causal agent of European ash dieback. Molecular Plant Pathology.

[12] McMullan M, Rafiqi M, Kaithakottil G, Clavijo BJ, Bilham L, Orton E, Percival-Alwyn L, Ward BJ, Edwards A, Saunders DGO (2018). The ash dieback invasion of Europe was founded by two genetically divergent individuals. Nature Ecology & Evolution.

[13] Inoue T, Okane I, Ishiga Y, Degawa Y, Hosoya T, Yamaoka Y (2019). The life cycle of *Hymenoscyphus fraxineus* on Manchurian ash, *Fraxinus mandshurica*, in Japan. Mycoscience.

[14] Gross A, Holdenrieder O: Pathogenicity of *Hymenoscyphus fraxineus* and *Hymenoscyphus albidus* towards *Fraxinus mandshurica* var. *japonica*. Forest Pathology 2015, 45(2):172–174.

[15] Gross A, Sieber TN (2016). Virulence of *Hymenoscyphus albidus* and native and introduced *Hymenoscyphus fraxineus* on *Fraxinus excelsior* and *Fraxinus pennsylvanica*. Plant Pathology.

[16] Kowalski T, Bilanski P, Holdenrieder O (2015). Virulence of *Hymenoscyphus albidus* and *H. fraxineus* on *Fraxinus excelsior* and *F. pennsylvanica*. PLoS One.

[17] Baral HO, Bemmann M (2014). *Hymenoscyphus fraxineus* vs. *Hymenoscyphus albidus* - a comparative light microscopic study on the causal agent of European ash dieback and related foliicolous, stroma-forming species. Mycology - An International Journal on Fungal Biology.

[18] Wey T, Schlegel M, Stroheker S, Gross A: MAT-gene structure and mating behavior of *Hymenoscyphus fraxineus* and *Hymenoscyphus albidus*. Fungal genetics and biology: FG & B 2016, 87:54–63. 10.1016/j.fgb.2015.12.013 26724599

[19] Stenlid J, Elfstrand M, Cleary M, Ihrmark K, Karlsson M, Davydenko K, Brandstrom-Durling M (2017). Genomes of *Hymenoscyphus fraxineus* and *Hymenoscyphus albidus* Encode Surprisingly Large Cell Wall Degrading Potential, Balancing Saprotrophic and Necrotrophic Signatures. Baltic Forestry.

[20] Dong S, Raffaele S, Kamoun S (2015). The two-speed genomes of filamentous pathogens: waltz with plants. Current Opinion in Genetics & Development.

[21] Stukenbrock EH, Croll D (2014). The evolving fungal genome. Fungal Biology Reviews.

[22] Slot JC, Gluck-Thaler E: Metabolic gene clusters, fungal diversity, and the generation of accessory functions. Current Opinion in Genetics & Development 2019, 58–59:17–24. 10.1016/j.gde.2019.07.006 31466036

[23] Castanera R, López-Varas L, Borgognone A, LaButti K, Lapidus A, Schmutz J, Grimwood J, Pérez G, Pisabarro AG, Grigoriev IV (2016). Transposable Elements versus the Fungal Genome: Impact on Whole-Genome Architecture and Transcriptional Profiles. PLOS Genetics.

[24] Muszewska A, Steczkiewicz K, Stepniewska-Dziubinska M, Ginalski K (2019). Transposable elements contribute to fungal genes and impact fungal lifestyle. Scientific Reports.

[25] Gioti A, Mushegian AA, Strandberg R, Stajich JE, Johannesson H (2012). Unidirectional evolutionary transitions in fungal mating systems and the role of transposable elements. Mol Biol Evol.

[26] Sun S, Yadav V, Billmyre RB, Cuomo CA, Nowrousian M, Wang L, Souciet J-L, Boekhout T, Porcel B, Wincker P (2017). Fungal genome and mating system transitions facilitated by chromosomal translocations involving intercentromeric recombination. PLoS biology.

[27] Badouin H, Hood ME, Gouzy J, Aguileta G, Siguenza S, Perlin MH, Cuomo CA, Fairhead C, Branca A, Giraud T (2015). Chaos of Rearrangements in the Mating-Type Chromosomes of the Anther-Smut Fungus *Microbotryum lychnidis-dioicae*. Genetics.

[28] Heitman J (2015). Evolution of sexual reproduction: A view from the fungal kingdom supports an evolutionary epoch with sex before sexes. Fungal Biology Reviews.

[29] Santana MF, Queiroz MV (2015). Transposable Elements in Fungi: A Genomic Approach. Scientific Journal of Genetics and Gene Therapy.

[30] Szitenberg A, Cha S, Opperman CH, Bird DM, Blaxter ML, Lunt DH (2016). Genetic Drift, Not Life History or RNAi, Determine Long-Term Evolution of Transposable Elements. Genome Biol Evol.

[31] Heitman J, Sun S, James TY (2013). Evolution of fungal sexual reproduction. Mycologia.

[32] Nellåker C, Keane TM, Yalcin B, Wong K, Agam A, Belgard TG, Flint J, Adams DJ, Frankel WN, Ponting CP (2012). The genomic landscape shaped by selection on transposable elements across 18 mouse strains. Genome Biol.

[33] Clutterbuck AJ (2011). Genomic evidence of repeat-induced point mutation (RIP) in filamentous ascomycetes. Fungal Genetics and Biology.

[34] Van de Wouw AP, Elliott CE, Popa KM, Idnurm A (2019). Analysis of Repeat Induced Point (RIP) Mutations in Leptosphaeria maculans Indicates Variability in the RIP Process Between Fungal Species. Genetics.

[35] Gioti A, Stajich JE, Johannesson H (2013). *Neurospora* and the dead-end hypothesis: genomic consequences of selfing in the model genus. Evolution; international journal of organic evolution.

[36] Ohm RA, Feau N, Henrissat B, Schoch CL, Horwitz BA, Barry KW, Condon BJ, Copeland AC, Dhillon B, Glaser F (2012). Diverse Lifestyles and Strategies of Plant Pathogenesis Encoded in the Genomes of Eighteen Dothideomycetes Fungi. PLoS Pathogens.

[37] Kristin L, Tesfaye M: Necrotroph Attacks on Plants: Wanton Destruction or Covert Extortion? The Arabidopsis Book 2010, 2010(8). 10.1199/tab.0136 PMC3244965 22303261

[38] Rokas A, Wisecaver JH, Lind AL (2018). The birth, evolution and death of metabolic gene clusters in fungi. Nature Reviews Microbiology.

[39] Andersson PF, Johansson SBK, Stenlid J, Broberg A (2010). Isolation, identification and necrotic activity of viridiol from *Chalara fraxinea*, the fungus responsible for dieback of ash. Forest Pathology.

[40] Andersson PF, Bengtsson S, Cleary M, Stenlid J, Broberg A (2013). Viridin-like steroids from *Hymenoscyphus pseudoalbidus*. Phytochemistry.

[41] Andersson PF, Bengtsson S, Stenlid J, Broberg A (2012). B-norsteroids from *Hymenoscyphus pseudoalbidus*. Molecules.

[42] Masi M, Di Lecce R, Tuzi A, Linaldeddu BT, Montecchio L, Maddau L, Evidente A (2019). Hyfraxinic Acid, a Phytotoxic Tetrasubstituted Octanoic Acid, Produced by the Ash (*Fraxinus excelsior* L.) Pathogen *Hymenoscyphus fraxineus* Together with Viridiol and Some of Its Analogues. Journal of Agricultural and Food Chemistry.

[43] Halecker S, Surup F, Kuhnert E, Mohr KI, Brock NL, Dickschat JS, Junker C, Schulz B, Stadler M (2014). Hymenosetin, a 3-decalinoyltetramic acid antibiotic from cultures of the ash dieback pathogen, *Hymenoscyphus pseudoalbidus*. Phytochemistry.

[44] Citron CA, Junker C, Schulz B, Dickschat JS (2014). A Volatile Lactone of *Hymenoscyphus pseudoalbidus*, Pathogen of European Ash Dieback, Inhibits Host Germination. Angewandte Chemie International Edition.

[45] Surup F, Halecker S, Nimtz M, Rodrigo S, Schulz B, Steinert M, Stadler M (2018). Hyfraxins A and B, cytotoxic ergostane-type steroid and lanostane triterpenoid glycosides from the invasive ash dieback ascomycete *Hymenoscyphus fraxineus*. Steroids.

[46] Halecker S, Wennrich J-P, Rodrigo S, Andrée N, Rabsch L, Baschien C, Steinert M, Stadler M, Surup F, Schulz B (2020). Fungal endophytes for biocontrol of ash dieback: The antagonistic potential of *Hypoxylon rubiginosum*. Fungal Ecology.

[47] Cleary MR, Andersson PF, Broberg A, Elfstrand M, Daniel G, Stenlid J (2014). Genotypes of *Fraxinus excelsior* with different susceptibility to the ash dieback pathogen *Hymenoscyphus pseudoalbidus* and their response to the phytotoxin viridiol - A metabolomic and microscopic study. Phytochemistry.

[48] Junker C, Mandey F, Pais A, Ebel R, Schulz B (2014). *Hymenoscyphus pseudoalbidus* and *Hymenoscyphus albidus*: viridiol concentration and virulence do not correlate. Forest Pathology.

[49] Simpson JT, Wong K, Jackman SD, Schein JE, Jones SJM, Birol İ (2009). ABySS: A parallel assembler for short read sequence data. Genome Research.

[50] Boetzer M, Henkel CV, Jansen HJ, Butler D, Pirovano W (2010). Scaffolding pre-assembled contigs using SSPACE. Bioinformatics.

[51] Li R, Zhu H, Ruan J, Qian W, Fang X, Shi Z, Li Y, Li S, Shan G, Kristiansen K *et al*: De novo assembly of human genomes with massively parallel short read sequencing. 2010, 20(2):265–272. 10.1101/gr.097261.109 PMC2813482 20019144

[52] Vezzi F, Narzisi G, Mishra B (2012). Reevaluating Assembly Evaluations with Feature Response Curves: GAGE and Assemblathons. PLoS ONE.

[53] Parra G, Bradnam K, Ning Z, Keane T, Korf I (2009). Assessing the gene space in draft genomes. Nucleic Acids Research.

[54] Holt C, Yandell M (2011). MAKER2: an annotation pipeline and genome-database management tool for second-generation genome projects. BMC Bioinformatics.

[55] Korf I (2004). Gene finding in novel genomes. BMC Bioinformatics.

[56] Stanke M, Morgenstern B: AUGUSTUS: a web server for gene prediction in eukaryotes that allows user-defined constraints. Nucleic Acids Research 2005, 33(Web Server issue):W465-W467. 10.1093/nar/gki458 PMC1160219 15980513

[57] Ter-Hovhannisyan V, Lomsadze A, Chernoff YO, Borodovsky M (2008). Gene prediction in novel fungal genomes using an ab initio algorithm with unsupervised training. Genome Research.

[58] Grabherr MG, Haas BJ, Yassour M, Levin JZ, Thompson DA, Amit I, Adiconis X, Fan L, Raychowdhury R, Zeng Q (2011). Full-length transcriptome assembly from RNA-Seq data without a reference genome. Nature biotechnology.

[59] Youssar L, Gruening BA, Erxleben A, Guenther S, Huettel W (2012). Genome Sequence of the Fungus *Glarea lozoyensis*: the First Genome Sequence of a Species from the Helotiaceae Family. Eukaryotic Cell.

[60] Gianoulis TA, Griffin MA, Spakowicz DJ, Dunican BF, Alpha CJ, Sboner A, Sismour AM, Kodira C, Egholm M, Church GM (2012). Genomic Analysis of the Hydrocarbon-Producing, Cellulolytic, Endophytic Fungus *Ascocoryne sarcoides*. PLoS Genetics.

[61] Zhu S, Cao Y-Z, Jiang C, Tan B-Y, Wang Z, Feng S, Zhang L, Su X-H, Brejova B, Vinar T (2012). Sequencing the genome of *Marssonina brunnea* reveals fungus-poplar co-evolution. BMC Genomics.

[62] Amselem J, Cuomo CA, van Kan JAL, Viaud M, Benito EP, Couloux A, Coutinho PM, de Vries RP, Dyer PS, Fillinger S (2011). Genomic Analysis of the Necrotrophic Fungal Pathogens *Sclerotinia sclerotiorum* and *Botrytis cinerea*. PLoS Genetics.

[63] Mardanov AV, Beletsky AV, Kadnikov VV, Ignatov AN, Ravin NV (2014). Draft genome sequence of *Sclerotinia borealis*, a psychrophilic plant pathogenic fungus. Genome Announcements.

[64] Spanu PD, Abbott JC, Amselem J, Burgis TA, Soanes DM, Stueber K, van Themaat EVL, Brown JKM, Butcher SA, Gurr SJ (2010). Genome Expansion and Gene Loss in Powdery Mildew Fungi Reveal Tradeoffs in Extreme Parasitism. Science.

[65] Staats M, van Kan JAL (2012). Genome Update of *Botrytis cinerea* Strains B05.10 and T4. Eukaryotic Cell.

[66] Lohse M, Nagel A, Herter T, May P, Schroda M, Zrenner R, Tohge T, Fernie AR, Stitt M, Usadel B (2014). Mercator: a fast and simple web server for genome scale functional annotation of plant sequence data. Plant, Cell & Environment.

[67] Bray N, Pachter L (2004). MAVID: Constrained Ancestral Alignment of Multiple Sequences. Genome Research.

[68] RepeatModeler Open-1.0. [http://www.repeatmasker.org]

[69] Margolin BS, Garrett-Engele PW, Stevens JN, Fritz DY, Garrett-Engele C, Metzenberg RL, Selker EU (1998). A methylated *Neurospora* 5S rRNA pseudogene contains a transposable element inactivated by repeat-induced point mutation. Genetics.

[70] Selker EU, Tountas NA, Cross SH, Margolin BS, Murphy JG, Bird AP, Freitag M (2003). The methylated component of the *Neurospora crassa* genome. Nature.

[71] Lewis ZA, Honda S, Khlafallah TK, Jeffress JK, Freitag M, Mohn F, Schuebeler D, Selker EU (2009). Relics of repeat-induced point mutation direct heterochromatin formation in *Neurospora crassa*. Genome Research.

[72] Ranwez V, Harispe S, Delsuc F, Douzery EJP (2011). MACSE: Multiple Alignment of Coding SEquences Accounting for Frameshifts and Stop Codons. PLoS ONE.

[73] Yang Z (2007). PAML 4: Phylogenetic Analysis by Maximum Likelihood. Molecular Biology and Evolution.

[74] Klopfenstein DV, Zhang L, Pedersen BS, Ramírez F, Warwick Vesztrocy A, Naldi A, Mungall CJ, Yunes JM, Botvinnik O, Weigel M (2018). GOATOOLS: A Python library for Gene Ontology analyses. Scientific Reports.

[75] Li L, Stoeckert CJ, Roos DS (2003). OrthoMCL: Identification of Ortholog Groups for Eukaryotic Genomes. Genome Research.

[76] De Bie T, Cristianini N, Demuth JP, Hahn MW (2006). CAFE: a computational tool for the study of gene family evolution. Bioinformatics.

[77] Weber T, Blin K, Duddela S, Krug D, Kim HU, Bruccoleri R, Lee SY, Fischbach MA, Müller R, Wohlleben W *et al*: antiSMASH 3.0—a comprehensive resource for the genome mining of biosynthetic gene clusters. Nucleic Acids Research 2015. 10.1093/nar/gkv437 PMC4489286 25948579

[78] Wang G-Q, Chen G-D, Qin S-Y, Hu D, Awakawa T, Li S-Y, Lv J-M, Wang C-X, Yao X-S, Abe I (2018). Biosynthetic pathway for furanosteroid demethoxyviridin and identification of an unusual pregnane side-chain cleavage. Nature Communications.

[79] Kakule TB, Sardar D, Lin Z, Schmidt EW (2013). Two Related Pyrrolidinedione Synthetase Loci in *Fusarium heterosporum* ATCC 74349 Produce Divergent Metabolites. ACS Chemical Biology.

[80] Kato N, Nogawa T, Hirota H, Jang J, Takahashi S, Ahn J, Osada HJB, communications br: A new enzyme involved in the control of the stereochemistry in the decalin formation during equisetin biosynthesis. 2015, 460 2:210–215. 10.1016/j.bbrc.2015.03.011 25770422

[81] Sir EB, Becker K, Lambert C, Bills GF, Kuhnert E (2019). Observations on Texas hypoxylons, including two new *Hypoxylon* species and widespread environmental isolates of the H. croceum complex identified by a polyphasic approach. Mycologia.

[82] Gilchrist CLM, Chooi Y-H: clinker & clustermap.js: automatic generation of gene cluster comparison figures. Bioinformatics 2021. 10.1093/bioinformatics/btab007 33459763

[83] de Wit PJGM, van der Burgt A, Okmen B, Stergiopoulos I, Abd-Elsalam KA, Aerts AL, Bahkali AH, Beenen HG, Chettri P, Cox MP *et al*: The Genomes of the Fungal Plant Pathogens *Cladosporium fulvum* and *Dothistroma septosporum* Reveal Adaptation to Different Hosts and Lifestyles But Also Signatures of Common Ancestry. Plos Genetics 2012, 8(11). 10.1371/journal.pgen.1003088 PMC3510045 23209441

[84] Arunkumar R, Ness RW, Wright SI, Barrett SCH (2015). The Evolution of Selfing Is Accompanied by Reduced Efficacy of Selection and Purging of Deleterious Mutations. Genetics.

[85] Takebayashi N, Morrell PL (2001). Is self-fertilization an evolutionary dead end? Revisiting an old hypothesis with genetic theories and a macroevolutionary approach. American Journal of Botany.

[86] Yun S-H, Berbee ML, Yoder OC, Turgeon BG: Evolution of the fungal self-fertile reproductive life style from self-sterile ancestors. Proceedings of the National Academy of Sciences 1999, 96(10):5592. 10.1073/pnas.96.10.5592 PMC21905 10318929

[87] Wright Stephen I, Ness Rob W, Foxe John P, Barrett Spencer CH (2008). Genomic Consequences of Outcrossing and Selfing in Plants. International Journal of Plant Sciences.

[88] Payne BL, Alvarez-Ponce D (2018). Higher Rates of Protein Evolution in the Self-Fertilizing Plant Arabidopsis thaliana than in the Out-Crossers Arabidopsis lyrata and Arabidopsis halleri. Genome Biology and Evolution.

[89] Charlesworth D, Wright SI (2001). Breeding systems and genome evolution. Current Opinion in Genetics & Development.

[90] McKinney LV, Thomsen IM, Kjaer ED, Bengtsson SBK, Nielsen LR (2012). Rapid invasion by an aggressive pathogenic fungus (*Hymenoscyphus pseudoalbidus*) replaces a native decomposer (*Hymenoscyphus albidus*): a case of local cryptic extinction?. Fungal Ecology.

[91] Halecker S, Surup F, Solheim H, Stadler M (2018). Albiducins A and B, salicylaldehyde antibiotics from the ash tree-associated saprotrophic fungus *Hymenoscyphus albidus*. The Journal of Antibiotics.

[92] Chen L, Yue Q, Zhang X, Xiang M, Wang C, Li S, Che Y, Ortiz-López FJ, Bills GF, Liu X (2013). Genomics-driven discovery of the pneumocandin biosynthetic gene cluster in the fungus *Glarea lozoyensis*. BMC Genomics.

[93] Moffatt JS, Bu’Lock JD, Yuen TH: Viridiol, a steroid-like product from *Trichoderma viride*. Journal of the Chemical Society D: Chemical Communications 1969(14):839a-839a.

[94] Reynolds HT, Slot JC, Divon HH, Lysøe E, Proctor RH, Brown DW (2017). Differential Retention of Gene Functions in a Secondary Metabolite Cluster. Molecular Biology and Evolution.

