# Supplementary material for: Comparative analyses of the Hymenoscyphus fraxineus and Hymenoscyphus albidus genomes reveals potentially adaptive differences in secondary metabolite and transposable element repertoires
Source: BMC Genomics. 2021 Jul 4;22:503. doi: 10.1186/s12864-021-07837-2 (PMC8254937; doi:10.1186/s12864-021-07837-2)
Supplement: Supplementary file 1 — Additional file 1: [file 12864_2021_7837_MOESM1_ESM.pdf]

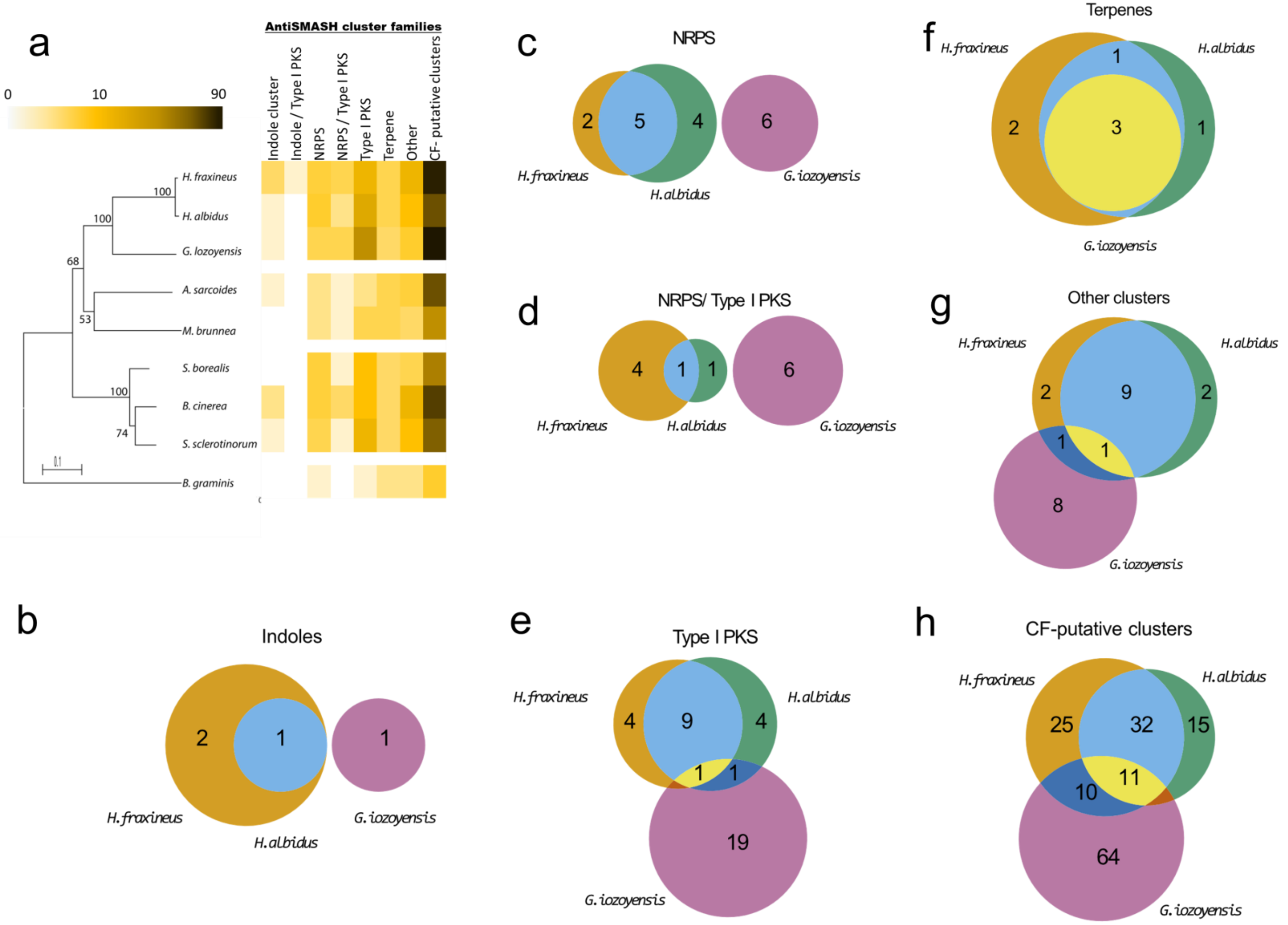

Supporting figure S1: Biosynthetic gene clusters (BGCs) predicted by AntiSMASH. a.) A heat-map of the number BGCs predicted in selected AntiSMASH families in *Hymenoscyphus fraxineus, Hymenoscyphus albidus, Glarea iozoyensis Ascocoryne sarcoides, Marssonina brunnea, Sclerotinia borealis, Botrytis cinerea, Sclerotinia sclerotiorum* and *Blumeria graminis* genomes annotated by our MAKER pipe-line. The Venn diagram in b-h show the overlap in identified clusters between *H. fraxineus, H. albidus, G. iozoyensis* in different AntiSMASH families. For a cluster to be considered to be overlapping in any species comparison species at least three genes in the clusters must be orthologous between species.
