# Supplementary material for: Comparative analyses of the Hymenoscyphus fraxineus and Hymenoscyphus albidus genomes reveals potentially adaptive differences in secondary metabolite and transposable element repertoires
Source: BMC Genomics. 2021 Jul 4;22:503. doi: 10.1186/s12864-021-07837-2 (PMC8254937; doi:10.1186/s12864-021-07837-2)
Supplement: Supplementary file 2 — Additional file 2: [file 12864_2021_7837_MOESM2_ESM.docx]

**Supplementary table S1.** Summary statistics for gene annotations in the studied species. Genes were predicted using the MAKER gene annotation pipeline.

|  | *H. fraxineus* | *H. albidus* | *G. lozoyensis* | *A. sarcoides* | *M. brunnea* | *S. borealis* | *S. sclerotiorum* | *B. cinerea* | *B. graminis* |
| --- | --- | --- | --- | --- | --- | --- | --- | --- | --- |
| Assembly length: | 64.2 Mb | 51.2 Mb | 39.5 Mb | 34.1 Mb | 51.9 Mb | 38.9 Mb | 38.3 Mb | 39.5 Mb | 111.3 Mb |
| Gap length: | 0.45 Mb | 0.42 Mb | 0.34 Mb | None | 0.23 Mb | 0.21 Mb | 0.33 Mb | 1.63 Mb | 30.8 Mb |
| G+C content: | 40 % | 44 % | 46 % | 46 % | 43 % | 42 % | 42 % | 43 % | 44 % |
| Longest scaffold: | 2.99 Mb | 0.61 Mb | 3.59 Mb | 1.15 Mb | 5.19 Mb | 0.59 Mb | 2.78 Mb | 1.47 Mb | 9.69 Mb |
| N50: | 997 kb (n:20) | 135 kb (n:108) | 2090 kb (n:8) | 395 kb (n:27) | 1600 kb (n:10) | 132 kb (n:86) | 1419 kb (n:9) | 510 kb (n:24) | 2063 kb (n:16) |
| Number of scaffolds > 1kb | 137 | 756 | 22 | 193 | 64 | 651 | 37 | 118 | 989 |
| CEGMA Complete | 96.4 % | 95.6 % | 96.4 % | 97.6 % | 94.8 % | 95.6 % | 95.6 % | 91.5 % | 94.0 % |
| CEGMA Partial | 97.2 % | 96.8 % | 98.0 % | 99.2 % | 96.4 % | 98.4 % | 98.4 % | 94.4 % | 97.2 % |
| Total transcripts | 14069 | 13947 | 13398 | 11358 | 9730 | 9249 | 10838 | 11246 | 6942 |
| - with GO assignments | 6509 | 6490 | 6698 | 5850 | 5063 | 5487 | 5614 | 5819 | 3669 |
| - with Pfam domains | 8205 | 8078 | 8474 | 7311 | 6379 | 6818 | 7001 | 7296 | 4545 |
| - with Uniprot hit | 5526 | 5453 | 5662 | 5051 | 4642 | 4917 | 4952 | 5077 | 3526 |
| - with splice est support | 7483 | 7538 | 0 | 0 | 0 | 0 | 2895 | 3927 | 821 |
| - with exon est support | 7225 | 7572 | 0 | 0 | 0 | 0 | 2823 | 3532 | 794 |
| - with est or protein evidence | 11305 | 11154 | 12885 | 10613 | 7503 | 9249 | 9548 | 9795 | 6219 |
| - with secretion signal (not transmembrane) | 2160 | 2006 | 1910 | 1256 | 1196 | 1094 | 1234 | 1376 | 896 |
| - short secreted proteins (<150 aa) | 308 | 294 | 140 | 99 | 119 | 48 | 80 | 93 | 145 |
