# Supplementary material for: Comparative analyses of the Hymenoscyphus fraxineus and Hymenoscyphus albidus genomes reveals potentially adaptive differences in secondary metabolite and transposable element repertoires
Source: BMC Genomics. 2021 Jul 4;22:503. doi: 10.1186/s12864-021-07837-2 (PMC8254937; doi:10.1186/s12864-021-07837-2)
Supplement: Supplementary file 6 — Additional file 6: [file 12864_2021_7837_MOESM6_ESM.docx]

Supplementary material: Comparative analyses of the Hymenoscyphus fraxineus and Hymenoscyphus albidus genomes reveals potentially adaptive differences in secondary metabolite and transposable element repertoires Elfstrand M., Chen J., Cleary M., Halecker S., Ihrmark K., Karlsson M., Davydenko K., Stenlid J., Stadler M., Brandström Durling M.

**Supplementary table S5.** Detailed information on the proposed members of the viridiol (*vir*) biosynthetic gene cluster (BGC). Presumed genes are located consecutively on scaffold 66 and scaffold 296 in the sequenced genomes of *H. fraxineus* nf4 and *H. albidus* 111/1/4, respectively.

| **Gene of viridiol BGC** | ***H. fraxineus* nf4 protein code** | ***H.albidus* 111/1/4 protein code** | **OMCL*** | **Deduced**  **function** | **Homologous gene of dimethoxyviridin BGC**  **in *Nodulisporium* sp. (size & accession no.)** |
| --- | --- | --- | --- | --- | --- |
| ***vir1*** | HYFRA _T00008107_1  (243 aa) | HYALB _T00010880_1  (243 aa) | omcl15095* | unknown | *vidN*  (254 aa, AVY05521) |
| ***vir2*** | HYFRA _T00008108_1  (310 aa) | HYALB _T00010879_1  (308 aa) | omcl15094* | Glyoxylase I | *vidQ*  (310 aa, AVY05524) |
| ***vir3*** | HYFRA _T00008109_1  (594 aa) | HYALB _T00010878_1  (595 aa) | omcl15238 | Cytochrome P450 monooxygenase | *vidR*  (547 aa, AVY05525) |
| ***vir4*** | HYFRA _T00008111_1  (496 aa) | HYALB _T00010877_1  (480 aa) | omcl15093* | Cytochrome P450 monooxygenase | *vidG*  (490 aa, AVY05514) |
| ***vir5*** | HYFRA _T00008112_1  (350 aa) | HYALB _T00010876_1  (350 aa) | omcl15092* | NADP-dependent dehydrogenase | *vidM*  (350 aa, AVY05520) |
| ***vir6*** | HYFRA _T00008113_1  (381 aa) | HYALB _T00010875_1  (381 aa) | omcl16084 | O-methyltransferase | *-* |
| ***vir7*** | HYFRA _T00008114_1  (579 aa) | HYALB _T00010874_1  (575 aa) | omcl10197 | Baeyer-Villiger monooxygenase | *vidF*  (541 aa, AVY05513) |
| ***vir8*** | HYFRA _T00008115_1  (521 aa) | HYALB _T00010873_1  (521 aa) | omcl17559 | Cytochrome P450 monooxygenase | *vidE*  (556 aa, AVY05512) |
| ***vir9*** | HYFRA _T00008116_1  (582 aa) | HYALB _T00010872_1  582 aa) | omcl8198* | Cytochrome P450 monooxygenase | *vidA,vidK,vidD*  (580 aa,578 aa, 601 aa;  AVY05508, AVY05518, AVY05511) |
| ***vir10*** | HYFRA _T00008117_1  (268 aa) | HYALB _T00010871_1  (152 aa) | omcl19821 | unknown (Bet v1-like protein) | *vidC, vidI*  (156 aa, 154 aa;  AVY05510, AVY05516) |
| ***vir11*** | HYFRA _T00008118_1  (321 aa) | HYALB _T00010870_1  (320 aa) | omcl15091* | Hydrolase | *vidP*  (321 aa, AVY05523) |
| ***vir12*** | HYFRA _T00008119_1  (286 aa) | HYALB _T00010869_1  (286 aa) | omcl15090* | NADP-dependent SDR | *vidH*  (283 aa, AVY05515) |
| ***vir13*** | - | HYALB _T00010868_1  (152 aa) | omcl19821 | unknown (Bet v1-like protein) | *vidC, vidI*  (156 aa, 154 aa;  AVY05510, AVY05516) |
| ***vir14*** | HYFRA _T00008120_1  (481 aa) | HYALB _T00010867_1  (482 aa) | omcl15089* | FAD-dependent oxidoreductase | *vidJ*  (505 aa, AVY05517) |
| ***vir15*** | HYFRA _T00008121_1  (252 aa) | HYALB _T00010866_1  (252 aa) | omcl9777 | NADP-dependent dehydrogenase | *vidO*  (253 aa, AVY05522) |
| ***vir16*** | HYFRA _T00008122_1  (464 aa) | HYALB _T00010881_1  (464 aa) | omcl15096* | FAD-dependent oxidoreductase | *-* |
| ***vir17*** | HYFRA _T00008123_1  (570 aa) | HYALB _T00010882_1  (606 aa) | omcl8198* | Cytochrome P450 monooxygenase | *vidA, vidD, vidK*  (580 aa, 601 aa, 578 aa,  AVY05508, AVY05511, AVY05518) |
| ***g18*** | HYFRA _T00008124_1  (565 aa) | HYALB _T00010883_1  (565 aa) | omcl4619 | unrelated | *-* |
| ***g19*** | HYFRA _T00008125_1  (394 aa) | HYALB _T00010884_1  (415 aa) | omcl7345 | Alpha/beta-Hydrolase | *-* |
| ***g20*** | HYFRA _T00008126_1  (569 aa) | HYALB _T00010885_1  (570 aa) | omcl4620 | unknown | *-* |
| ***g21*** | HYFRA _T00008127_1  (351 aa) | HYALB _T00010886_1  (350 aa) | omcl4618 | Alpha/beta-Hydrolase | *-* |

OMCL families marked with an asterisk (*) are unique to the *Hymenoscyphus* branch
