# Supplementary material for: Comparative analyses of the Hymenoscyphus fraxineus and Hymenoscyphus albidus genomes reveals potentially adaptive differences in secondary metabolite and transposable element repertoires
Source: BMC Genomics. 2021 Jul 4;22:503. doi: 10.1186/s12864-021-07837-2 (PMC8254937; doi:10.1186/s12864-021-07837-2)
Supplement: Supplementary file 7 — Additional file 7: [file 12864_2021_7837_MOESM7_ESM.docx]

Supplementary material: Comparative analyses of the *Hymenoscyphus fraxineus* and *Hymenoscyphus albidus* genomes reveals potentially adaptive differences in secondary metabolite and transposable element repertoires: Elfstrand M., Chen J., Cleary M., Halecker S., Ihrmark K., Karlsson M., Davydenko K., Stenlid J., Stadler M., Brandström Durling M.

**Supplementary table S6.** Details on the proposed members of the hymenosetin (*hym*) BGC. Presumed genes are located consecutively on scaffold 52 in the sequenced genome of *H. fraxineus* nf4*.*

| **Gene of hymenosetin BGC**  ***in H. fraxineus* nf4** | ***H. fraxineus* nf4 protein code** | **OMCL** | **Deduced**  **function** | **Homologous gene of equisetin BGC**  **in *Fusarium heterosporum*.** |
| --- | --- | --- | --- | --- |
| ***hymS***  (4008 aa) | HYFRA _T00011526_1 | omcl1001 | PKS-NRPS hybrid | *eqxS*  (3948 aa, AGO86662) |
| ***hymA***  (388 aa) | HYFRA _T00011527_1 | N/A | Diels Alderase | *eqx3*  (377 aa, AGO86663) |
| ***hymC***  (353 aa) | HYFRA _T00011528_1 | omcl9982 | Trans-  enoyl reductase | *eqxC*  (353 aa, AGO86659) |
| ***hymG***  (491 aa) | HYFRA _T00011529_1 | omcl1371 | MFS transporter | *eqxG*  (481 aa AGO86666) |
| ***hymF***  (816 aa) | HYFRA _T00011530_1 | omcl9210 | Transcription factor | *eqxF*  (696 aa, AGO86660) |
| ***hymR***  (412 aa) | HYFRA _T00011531_1 | omcl14521 | Transcription factor | *eqxR*  (469 aa AGO86667) |
